# Early Developmental PMCA2b Expression Protects From Ketamine-Induced Apoptosis and GABA Impairments in Differentiating Hippocampal Progenitor Cells

**DOI:** 10.3389/fncel.2022.890827

**Published:** 2022-05-23

**Authors:** Malwina Lisek, Joanna Mackiewicz, Marta Sobolczyk, Bozena Ferenc, Feng Guo, Ludmila Zylinska, Tomasz Boczek

**Affiliations:** ^1^Department of Molecular Neurochemistry, Medical University of Lodz, Łódz, Poland; ^2^Department of Pharmaceutical Toxicology, China Medical University, Shenyang, China

**Keywords:** ketamine, GABA metabolism, hippocampal progenitor cells, plasma membrane Ca^2+^-ATPase (PMCA), calcium, neuronal differentiation

## Abstract

PMCA2 is not expressed until the late embryonic state when the control of subtle Ca^2+^ fluxes becomes important for neuronal specialization. During this period, immature neurons are especially vulnerable to degenerative insults induced by the N-methyl-D-aspartate (NMDA) receptor blocker, ketamine. As H19-7 hippocampal progenitor cells isolated from E17 do not express the PMCA2 isoform, they constitute a valuable model for studying its role in neuronal development. In this study, we demonstrated that heterologous expression of PMCA2b enhanced the differentiation of H19-7 cells and protected from ketamine-induced death. PMCA2b did not affect resting [Ca^2+^]_c_ in the presence or absence of ketamine and had no effect on the rate of Ca^2+^ clearance following membrane depolarization in the presence of the drug. The upregulation of endogenous PMCA1 demonstrated in response to PMCA2b expression as well as ketamine-induced PMCA4 depletion were indifferent to the rate of Ca^2+^ clearance in the presence of ketamine. Yet, co-expression of PMCA4b and PMCA2b was able to partially restore Ca^2+^ extrusion diminished by ketamine. The profiling of NMDA receptor expression showed upregulation of the NMDAR1 subunit in PMCA2b-expressing cells and increased co-immunoprecipitation of both proteins following ketamine treatment. Further microarray screening demonstrated a significant influence of PMCA2b on GABA signaling in differentiating progenitor cells, manifested by the unique regulation of several genes key to the GABAergic transmission. The overall activity of glutamate decarboxylase remained unchanged, but Ca^2+^-induced GABA release was inhibited in the presence of ketamine. Interestingly, PMCA2b expression was able to reverse this effect. The mechanism of GABA secretion normalization in the presence of ketamine may involve PMCA2b-mediated inhibition of GABA transaminase, thus shifting GABA utilization from energetic purposes to neurosecretion. In this study, we show for the first time that developmentally controlled PMCA expression may dictate the pattern of differentiation of hippocampal progenitor cells. Moreover, the appearance of PMCA2 early in development has long-standing consequences for GABA metabolism with yet an unpredictable influence on GABAergic neurotransmission during later stages of brain maturation. In contrast, the presence of PMCA2b seems to be protective for differentiating progenitor cells from ketamine-induced apoptotic death.

## Introduction

The functional specialization of developing neurons requires specific demands with respect to the Ca^2+^ handling toolkit, which must respond to local changes in Ca^2+^ signaling in different compartments of each neuron. Spatial and temporal control of Ca^2+^ signals is required for the specification of neurotransmitter subtypes, dendritic growth and arborization, and axon pathfinding (Rosenberg and Spitzer, 2011). The generation of discrete subcellular Ca^2+^ transients is consistent with the idea that local activity patterns shape the influence of Ca^2+^ on neuronal development. In this process, the importance of Ca^2+^ extrusion systems is equal to the influx systems, and disruption of this equilibrium may have detrimental consequences on the functional properties of an immature neuron. During embryonic development, hippocampal neurons undergo a plethora of dynamic changes associated with active remodeling and the formation of new synaptic connections (Urbán and Guillemot, 2014; Hayashi et al., 2015; Cossart and Khazipov, 2022). During this period, the maintenance of a resting cytosolic Ca^2+^ concentration ([Ca^2+^]_c_) and its restoration following Ca^2+^ elevations falls upon plasma membrane Ca^2+^-ATPase (PMCA) isoforms 1 and 4. Two other PMCA isoforms 2 and 3 are expressed later in development and are highly upregulated postnatally. For instance, PMCA2 is induced on day 18 of embryonic development and is concentrated at high levels in the adult hippocampus and cerebellum (Marcos et al., 2009; Mata and Sepulveda, 2010). It is believed that the late appearance of this isoform may be relevant to spatially confined Ca^2+^ signaling that underlies the specificity of synaptic function (Augustine et al., 2003). Thus, the control of [Ca^2+^]_c_ elevations in early development will be achieved through transcriptional and/or activity regulation of PMCA1 and PMCA4. This is of paramount importance for the Ca^2+^-mediated specification of the GABAergic phenotype. It has been demonstrated that Ca^2+^ spiking is necessary and sufficient to drive the expression of GABA machinery (Holliday et al., 1991; Gu and Spitzer, 1995; Rosenberg and Spitzer, 2011). Attenuation of spontaneous Ca^2+^ oscillatory activity reduced the number of GABAergic cells, further indicating a relationship between regulation of GABA expression and spiking activity. However, the role of PMCA-dependent [Ca^2+^]_c_ regulation in GABA function during development remains unknown.

In immature neurons and neural progenitors, GABA depolarizes the plasma membrane and acts in synergy with N-methyl-D-aspartate (NMDA) signaling (Ben-Ari, 2002; Wang and Kriegstein, 2008). The presence of active NMDA receptors (NMDAR) sensitizes developing neurons to neurotoxic insults induced by NMDAR blockers, including the recreationally and clinically used drug, ketamine. It has been demonstrated that ketamine led to massive apoptotic neurodegeneration in the neonatal brain that is restricted exclusively to neurons (Ikonomidou et al., 1999). Increased apoptotic death was demonstrated to coincide with suppression of spontaneous oscillatory activity and irreversible loss of intracellular Ca^2+^ homeostasis (Sinner et al., 2011). An increasing body of evidence further supports the calcium hypothesis of ketamine neurotoxicity, suggesting several potential compensatory mechanisms that may lead to the accumulation of intracellular Ca^2+^ to potentially toxic concentrations (Shi et al., 2010; Liu et al., 2013; Wang et al., 2017; Lisek et al., 2020). Although no reports are available for immature neurons, data from the adult brain suggest that ketamine may interfere with the assembly of local NMDAR/PSD-95/PMCA densities at the synaptic membrane, concentrating PMCA within Ca^2+^ entry zones (Lisek et al., 2017). In contrast, ketamine has been shown to affect the expression of genes encoding main players in neuronal Ca^2+^ handling (Lisek et al., 2016) and directly inhibit PMCA activity (Boczek et al., 2015b). As the inhibitory action on PMCA was not specific to a particular isoform, we can assume that a similar mechanism may also exist in progenitor and immature cells. Because Ca^2+^ rises are a well-known trigger for GABA release (Haugstad et al., 1992), it is not surprising that any protracted disruptions in [Ca^2+^]_c_ may result in the aberrant function of the GABAergic system during early development. In this study, we used PMCA2-GFP-expressing H19-7 cells to study how PMCA2 affects the phenotype of differentiating neurons and whether it may preserve GABA synthesis and secretion in the presence of ketamine. H19-7 cells have been isolated from E17 of the rat hippocampus and conditionally immortalized by the expression of SV40 large T antigen (Eves et al., 1992). Because of their properties enabling propagation while they are immortalized and differentiation in the absence of an immortalization signal as well as the developmental absence of the PMCA2 isoform, they are a well-suited model to study the role of this calcium pump in the differentiation of hippocampal neurons.

## Materials and Methods

### Reagents

All reagents, if not separately mentioned, were purchased from Merck (Germany). Ketamine (in the form of ketamine hydrochloride) was purchased from Biowet (Poland). BCIP/NBT tablets were purchased from Merck (Germany). Protein Assay Kit was purchased from Bio-Rad (USA). Rabbit polyclonal anti-PMCA1 (Cat. No. PA1-914), rabbit polyclonal anti-PMCA2 (Cat. No. PA1-915), rabbit polyclonal anti-PMCA3 (Cat. No. PA1-916), mouse monoclonal anti-PMCA4 (Cat. No. MA1-914), rabbit polyclonal anti-NMDAR1 (Cat. No. PA3-102), rabbit polyclonal anti-NMDAR2A (Cat. No. A-6473), rabbit polyclonal anti-NMDAR2B (Cat. No. PA3-104), anti-NF68 (Cat. No. MA5-14981), anti-GAP43 (Cat. No. PA5-34943), normal mouse IgG1, normal rabbit IgG as well as secondary antibodies conjugated with alkaline phosphatase were purchased from Thermo Scientific (USA). Anti-phospho-Bad (Ser-136) antibodies (Cat. No. 9295) and antibodies recognizing total Bad protein (Cat. No. 9292) were purchased from Cell Signaling Technology (USA). Primers were synthesized in the Institute of Biochemistry and Biophysics (Poland). GABA release kit was purchased from Cloud-Clone Corp. (USA).

### H19-7 Cell Culture and Transfection

H19-7/IGF-IR (ATCC CRL-2526) cell lines derived from rat hippocampi dissected on embryonic day 17 were grown in plates pre-coated with 0.015 mg/ml poly-L-lysine, in Dulbecco's Modified Eagle's Medium (DMEM) supplemented with 0.2 mg/ml G418, 0.001 mg/ml puromycin and 10% fetal bovine serum (FBS) in a humidified incubator at 34°C. To obtain a neuronal phenotype, cells were moved to an incubator at 39°C and grown in DMEM containing 10 ng/ml basic fibroblast growth factor (bFGF), N2 supplement, 1% FBS, and 274 μM ketamine (treated cells) or saline (control) for 48 h. Ketamine at an IC_50_ value of 274 μM has been demonstrated to reduce calcium current in H19-7 cells (Huang et al., 2012).

PMCA2b or PMCA4b expressing H19-7 line was obtained after transfection with pEGFP-PMCA2b or mCherry-PMCA4b eukaryotic vectors using Lipofectamine LTX, yielding an efficiency of 70–80%. The expression level of PMCA2b or PMCA4b was checked by real-time PCR and Western blot 48 h later. H19-7 cells transfected with empty GFP and/or mCherry plasmid were used as a control. PMCA1 silencing was obtained using siRNA (20 nM final concentration) delivered with RNAiMAX according to the protocol provided by the manufacturer. The efficiency of silencing was evaluated using real-time PCR and Western blotting 48 h after. Scrambled siRNA was used as a control. The morphological analysis of neurite outgrowth was done using ImageJ with Simple Neurite Tracer. The length of neurites for at least 15 cells per condition was measured in triplicate. The protrusion was defined as a neurite when its length was at least double the cell body diameter. For quantification, only morphologically intact neurons were included.

### Viability Assay

The number of cells in apoptosis or necrosis was counted using FACScan Becton Dickinson and analyzed using CELLQuest Becton Dickinson software. Cells were stained with Annexin V/propidium iodide using the Annexin V-FITC Apoptosis Detection Kit I according to the manufacturer's instructions. Briefly, cells were harvested and washed two times with ice-cold PBS and incubated with FITC Annexin V and propidium iodide for 15 min at 25°C in the dark.

### RNA Isolation and Real-Time PCR

Total RNA was isolated with Trizol^®^ Reagent according to the manufacturer's instructions. For single-stranded cDNA synthesis, 1 μg of extracted RNA and oligo(dT) primers with M-MLV reverse transcriptase were used. The cDNA was used as a template for a real-time PCR performed with Maxima SYBR Green Master Mix and detected by the AbiPrism 7000 system (Applied Biosciences) under the following conditions: 15 min of initial activation at 95°C, followed by 40 cycles at 95°C for 15 s, 60°C for 30 s, and 72°C for 30 s. After every experiment, a melting curve was created. The data were analyzed using the 2^−Δ*Δct*^ method (Livak and Schmittgen, 2001), with Gapdh as reference housekeeping control, and showed a relative fold change. The used primers are listed in Table 1.

**Table 1:** Primers used in the real-time reaction.

**Gene**	**Protein**	**Primer sequence**
*Atp2b1*	PMCA1	F: 5'-CCTGAGGTACCAGAGGCAATAAA-3'
		R: 5'-TGGGTGTAAAATACCGCATTTG-3'
*Atp2b2*	PMCA2	F: 5'-ACCGTGGTGCAGGCCTATGT-3'
		R: 5'-GGCAATGGCGTTGACCAGCA-3'
*Atp2b4*	PMCA4	F: 5'-ACGCGGTGTATCAGCTCGGA-3'
		R: 5'-AGTGCTGGCTGGGTGGTGAA-3'
*Grin1*	NMDAR1	F: 5'-GCTGTACCTGCTGGACCGCT-3'
		R: 5'-GCAGTGTAGGAAGCCACTATGATC-3'
*Grin2a*	NMDAR2a	F: 5' -GCTACGGGCAGACAGAGAAG-3'
		R: 5' -GTGGTTGTCATCTGGCTCAC-3'
*Grin2b*	NMDAR2b	F: 5'-GCTACAACACCCACGAGAAGAG-3'
		R: 5'-GAGAGGGTCCACGCTTTCC-3'
*Gapdh*	GAPDH	F: 5'-GGTTACCAGGGCTGCCTTCT-3'
		R: 5'-CTTCCCATTCTCAGCCTTGACT-3'

### Microarray Screening

The expression of 84 genes involved in GABA and glutamate signaling was detected using RT^2^ Profiler PCR Array (PARN-152Z). The reaction mixture, based on RT^2^ SYBR Green qPCR Mastermix and cDNA synthesized from 2 μg of RNA (isolated as described in RNA isolation and rtPCR), was added to a 96-well plate (25 μl per well). Thermal cycling was carried out on the AbiPrism 7000 System (Applied Biosciences) under the following conditions: stage 1: 10 min of initial activation at 95°C; stage 2: 40 cycles of 95°C for 15 s and 60°C for 1 min; stage 3: 95°C for 1 min, 55°C for 30 s, and 95°C for 30 s (dissociation curve). Data were presented as a fold change calculated using the 2^−Δ*ΔCt*^ method (Livak and Schmittgen, 2001)—normalized gene expression (2^−Δ*Ct*^) in the test sample divided by the normalized gene expression (2^−Δ*Ct*^) in the control sample. The housekeeping genes used for normalization were chosen according to recommendations by Vandesompele et al. (2002). Statistical analyses were made with RT^2^ Profiler PCR Array data analysis software version 3.5 (SABiosciences).

### Western Blotting

Proteins were extracted in RIPA buffer supplemented with PMSF and an inhibitor cocktail. The total protein level was quantified colorimetrically with the Bio-Rad Protein Kit Assay. Protein samples were separated on SDS-PAGE and transferred to a nitrocellulose membrane (for details see Boczek et al., 2015a). Membranes were incubated overnight with primary antibodies: PMCA1 (1:1,000), PMCA2 (1:1,000), PMCA3 (1:1,000), PMCA4 (1:1,000), NMDAR1 (1:1,000), NMDAR2A (1:1,000), NMDAR2B (1:1,000), GAP43 (1:1,000), NF68 (1:1,000), Bad (1:1,000), p-Bad Ser-136 (1:500), and GAPDH (1:1,000). After three washes with TBS-T buffer and 2 h of incubation with secondary antibodies conjugated with alkaline phosphatase (AP), membranes were developed using the BCIP/NBT solution. Blots were scanned, and the band intensity was analyzed densitometrically using ImageJ, version 1.49 (NIH, Bethesda, MD, USA). The results were expressed as arbitrary units (AU) obtained after normalization to the endogenous GAPDH level.

### Enzyme Activity Assays

The determination of GAD activity was done fluorometrically according to the method described previously (Graham and Aprison, 1966). In brief, cell lysates (~5 mg/ml protein) were added to reaction buffer (25 mM glutamate, 0.5 mM pyridoxal phosphate, 0.1% Triton X-100, 200 mM Na_2_HPO_4_, 200 mM NaH_2_PO_4_, and pH 6.4) in a 1:1 ratio and incubated at 37°C for 30 min. To stop the reaction, trichloroacetic acid was added at a final concentration of 5%. In the next step, the samples were mixed with ninhydrin in carbonate-bicarbonate buffer and incubated at 60°C for 20 min. After dilution with a cooper-tartrate solution in a 1:13 ratio and a 20-min incubation, the fluorescence of samples was measured at 470 nm using an excitation wavelength of 365 nm by Perkin Elmer LS-50B spectrofluorometer (Perkin Elmer, USA). The GAD activity was presented as nmol GABA/mg protein/ml.

GABA-T activity measure was based on the modified coupled method with NAD^+^ (De Boer and Bruinvels, 1977). Cell lysates (~10 mg/ml protein) were mixed with a buffer containing 0.67% Triton X-100, 50 mM Na_4_P_2_O_7_, pH 8.5, and 4.5 mM 2-mercaptoethanol in a 1:1 ratio and incubated on ice for 1 h. Next, samples were mixed in a 1:5 ratio with reaction buffer (2 mM α-ketoglutarate, 3 mM GABA, 1 mM NAD^+^ in pyrophosphate buffer, pH 8.5) and incubated at 37°C for 1 h and then stopped by rapid cooling to 4°C. The formation of NAD^+^ was determined at 340 nm using a plate reader (BioTek, USA). GABA-T activity was presented as nmol NADH/mg protein/min.

### Single-Cell Calcium Imaging

Cells grown on poly-l-lysine coated coverslips were loaded with Fluo-4 calcium indicator for 30 min. The measurements were taken using a Leica DMi8 inverted microscope with an accompanying system. After excitation at 488 nm, images from emission at 520 nm were collected. The values gained from the region of interest (ROI) were used to calculate resting and stimulated [Ca^2+^]_i_. The cells did not change in size, shape, or location during the experiments. R_max_ was obtained by adding 10 μM ionomycin in the presence of 10 mM CaCl_2_, and R_min_ was determined by adding 10 mM EGTA in a Ca^2+^-free buffer.

### Quantification of GABA Release

The release of GABA into the culture medium was measured using Enzyme-Linked Immunosorbent Assay Kit for GABA according to the manufacturer's instruction. Briefly, H19-7 cells were washed 2 times with Locke's buffer (pH 7.4) and subsequently stimulated with high potassium (30 mM) Locke's buffer for 5 min. The cell debris was separated by brief centrifugation, and the culture medium was collected. The absorbance was read at 450 nm using a Victor X3 fluorescence plate reader (Perkin Elmer, USA). GABA release was calculated based on a simultaneously prepared calibration curve and was expressed as pmoles/mg/min following normalization to the protein content. The release experiment was carried out at a temperature of 25 ± 1°C.

### Statistical Analysis

The data are presented as mean ± SEM of number of experiments (*n* ≥ 3). Statistical analyses were made using STATISTICA 8.0 (StatSoft). For multiple comparisons, ANOVA with Tukey's correction was used. A *P*-value of <0.05 was considered statistically significant.

## Results

### Heterologous PMCA2b Expression Accelerates Differentiation of H19-7 Cells and Protects From Ketamine-Induced Apoptotic Death

**Figure 1:**
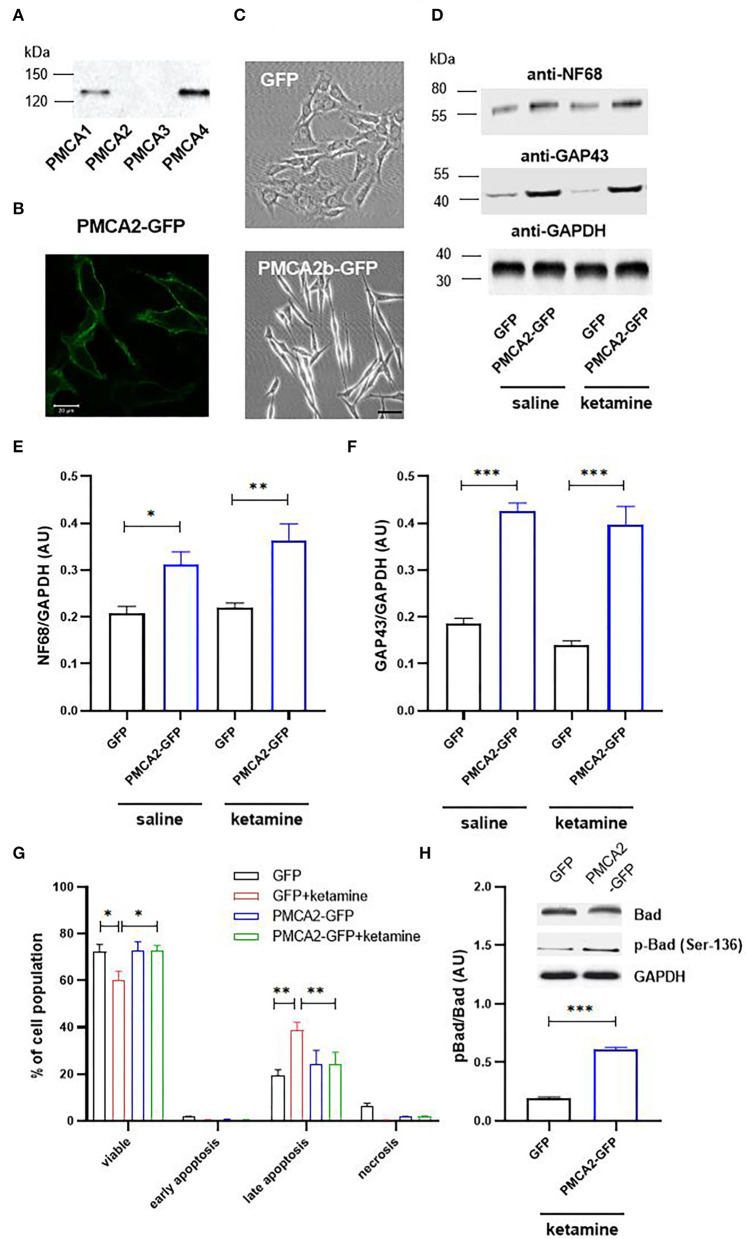
PMCA2b expression enhances H19-7 cell differentiation and restricts ketamine-mediated apoptosis. **(A)** The presence of PMCA isoforms determined using the Western blot. **(B)** The expression of PMCA2b-GFP and the targeting of the fusion protein to the plasma membrane visualized using immunocytochemistry. Scale bar 20 μm. **(C)** The effect of PMCA2b expression on cellular morphology in the absence of a differentiating agent. Scale bar 50 μm. **(D)** The protein level of sdifferentiation markers, NF68 and GAP43, determined using the Western blot. For **(A–D)**, the representative images are presented. **(E)** Quantification of NF68 and **(F)** GAP43 band intensity following normalization to endogenous Gapdh level, *n* = 4. AU, arbitrary units. **(G)** Flow cytometry analysis of differentiated cell viability in the presence or absence of ketamine. **(H)** Quantification of Bad phosphorylation at Ser-136. Total Bad and p-Bad Ser-136 protein level determined by the Western blot in the presence of ketamine was normalized to Gapdh, and the results are presented as phosphorylation index (pBad/Bad), *n* = 3. Representative blots are shown. *P < 0.05, **P < 0.01, ***P < 0.001.

First, we determined the expression pattern of PMCA isoforms in progenitor H19-7 cells. As shown in Figure 1A, the immunoreactivity for PMCA1 and PMCA4 was detected, whereas PMCA2 and PMCA3 were absent. This is consistent with other reports as well as developmental regulation of PMCA isoforms showing the appearance of PMCA2 and PMCA3 after E18 (Strehler and Zacharias, 2001; Bhargava et al., 2002). We next expressed PMCA2b fused to GFP and demonstrated that the majority of GFP-tagged protein was effectively targeted to the plasma membrane (Figure 1B). PMCA2b expression was sufficient to induce the formation of short neurite-like protrusions in the absence of any differentiating agents, indicating early stages of neuritogenesis (Figure 1C). Following a 2-day differentiation period in the presence of basic fibroblast growth factor (bFGF), PMCA2b-expressing cells demonstrated significantly higher levels of neuronal markers, namely, NF68 and GAP-43, compared to GFP control (Figures 1D–F). Measurement of the longest neurite per cell revealed that PMCA2b expression increased axon extension compared with GFP control (223 ± 28 μm vs. 168 ± 19 μm in GFP control). No changes in the expression of NF68 or GAP-43 and the dynamics of the differentiation process were seen following ketamine treatment. The viability of differentiating cells was assessed by double staining with Annexin V and propidium iodide (Figure 1G). In line with other reports (Eves et al., 1998; Pfisterer and Khodosevich, 2017), we observed increased apoptosis, which was enhanced in GFP-control cells in the presence of ketamine, but the expression of PMCA2b rescued neurons from ketamine-induced death. Simultaneously, we observed higher phosphorylation of pro-apoptotic protein Bad in PMCA2b-expressing cells cultured in the presence of the drug (Figure 1H).

### PMCA2b Increases Ca^2+^ Clearance Following KCl Stimulation in Saline but Not in Ketamine-Treated Cells

**Figure 2:**
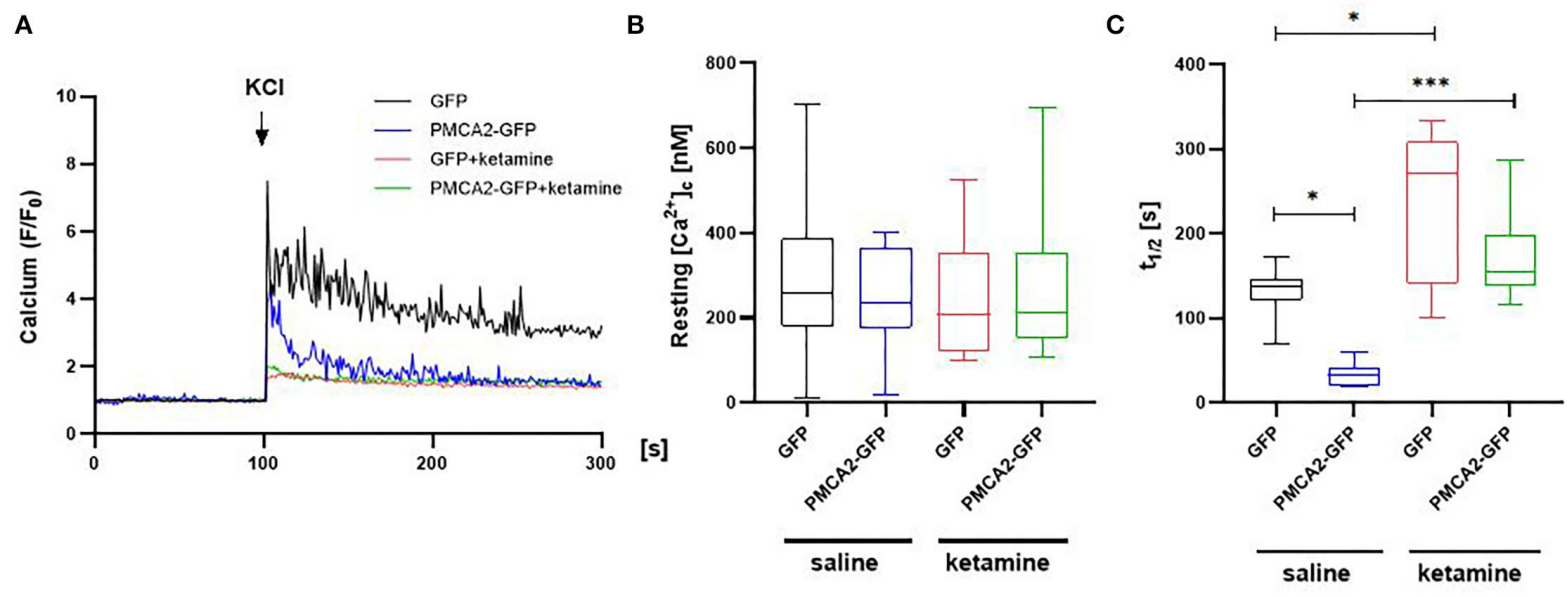
The effect of PMCA2b expression on KCl-induced Ca^2+^ transients in the presence or absence of ketamine. **(A)** Representative traces of Fluo-4 fluorescence intensity (F/F_0_) changes in response to 30 mM KCl pulse measured in single cells using Leica DMi8 inverted microscope. **(B)** Quantification of a resting Ca^2+^ in cytosol ([Ca^2+^]_c_), *n* = 17. **(C)** Halftime of Ca^2+^ signal decay, *n* = 17. For **(B,C)**, the data are plotted from max to min with the line centered at the mean. *P < 0.05, ***P < 0.001.

To check if PMCA2b expression was associated with a physiologically significant effect on [Ca^2+^]_c_, we performed single-cell imaging using a single-wavelength Ca^2+^ indicator, Fluo4. Stimulation with 30 mM KCl in the presence or absence of ketamine showed a PMCA2- and/or drug-dependent response (Figure 2A). However, neither ketamine nor GFP control or PMCA2-GFP affected the pre-stimulus resting calcium concentration ([Ca^2+^]_c_) as shown in Figure 2B. In the saline-treated group, PMCA2b-GFP expression decreased the halftime of Ca^2+^ signal decay (t_1/2_) from 131 ± 11 s to 33 ± 5 s (Figure 2C). No similar effect of PMCA2b was seen in ketamine-treated cells, which exhibited a substantial delay in Ca^2+^ clearance.

### Endogenous PMCA1 and PMCA4 Do Not Significantly Participate in Enhanced Ca^2+^ Clearance in PMCA2b-Expressing Cells

**Figure 3:**
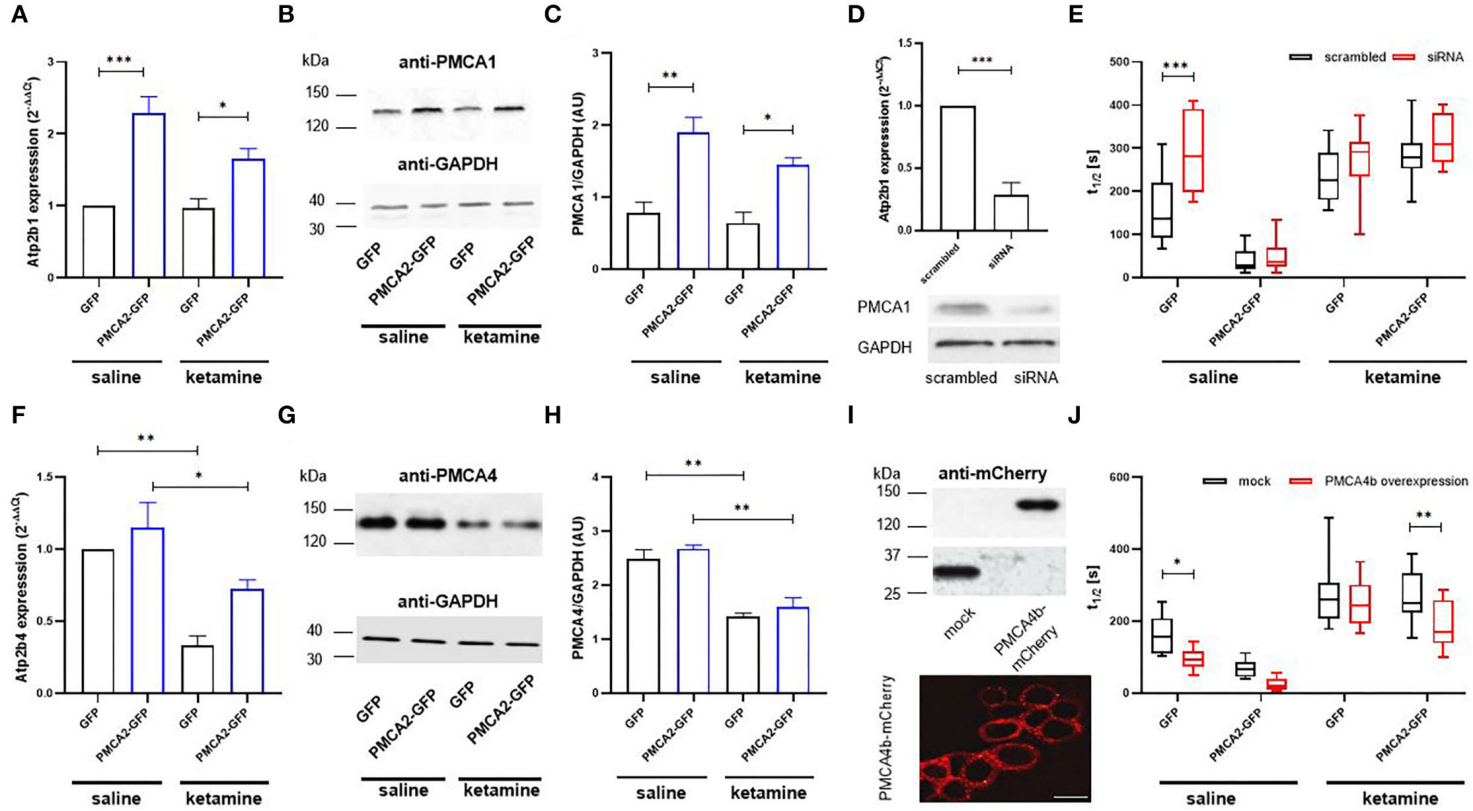
The contribution of PMCA isoforms to the rate of Ca^2+^ clearance following 30 mM KCl treatment in the presence or absence of ketamine. **(A)** The expression of *Atp2b1* in GFP- or PMCA2b-GFP expressing cells was qualified using real-time PCR. The raw data were normalized to endogenous *Gapdh* expression and were calculated based on the 2^−Δ*ΔCt*^ method to obtain relative fold change. The expression level in GFP control treated with saline was taken as 1, *n* = 4. **(B)** PMCA1 protein level was determined with the Western blot. GAPDH was used as a loading control. The representative images are shown. **(C)** Quantification of PMCA1 protein level following normalization to endogenous Gapdh level, *n* = 3. AU, arbitrary units. **(D)** The efficiency of *Atp2b1* silencing with siRNA evaluated using real-time PCR and confirmed at the protein level with the Western blot. The reduction in mRNA level was quantified using the 2^−Δ*ΔCt*^ method, and the expression in scrambled-treated cells was taken as 1, *n* = 3. **(E)** Halftime of signal decay (t_1/2_) of individual tracings recorded from scrambled or PMCA1 siRNA-treated cells, presented as interleaved box graph centered at the mean. **(F)** The expression of *Atp2b4* in GFP- or PMCA2b-GFP expressing cells, *n* = 3. **(G)** Immunoreactivity of PMCA4 determined with the Western blot. **(H)** Quantification of PMCA4 protein level following normalization to endogenous Gapdh level, *n* = 3. AU, arbitrary units. **(I)** Over-expression of PMCA4b-mCherry protein and the targeting of the fusion protein to the plasma membrane visualized by immunocytochemistry. Scale bar 20 μm. **(J)** Halftime of signal decay (t_1/2_) of individual tracings recorded from mCherry- or PMCA4b-mCherry-positive cells, presented as interleaved box graph centered at the mean. Here, the mock-transfected control was cells double transfected with GFP and mCherry. *P < 0.05, **P < 0.01, ***P < 0.001.

To verify if PMCA2b was solely responsible for the enhanced Ca^2+^ clearance following a KCl pulse, we first searched for any changes within the PMCA1 and PMCA4 isoforms. The expression profiling using real-time PCR showed a 2.3 × higher message for *Atp2b1* (encoding PMCA1) in PMCA2b-positive cells (Figure 3A), and this effect persisted in ketamine-treated cells. The changes in the mRNA were also seen at the level of PMCA1 protein (Figures 3B,C). To determine the contribution of PMCA1 to enhanced Ca^2+^ clearance in a PMCA2b-expressing line, RNAi was used to selectively decrease *Atp2b1* expression (Figure 3D). We first established that the transfection of PMCA1-specific siRNA resulted in a 71 ± 11% decrease in *Atp2b1* expression. Concomitantly, we detected a partial loss of PMCA1 immunoreactivity. In such circumstances, t_1/2_ increased by 132 ± 26 s in the siRNA-treated GFP control, while no changes in the rate of Ca^2+^ clearance were seen between scrambled or siRNA-treated PMCA2b-expressing cells (Figure 3E). PMCA1 silencing did not affect t_1/2_ in any of the groups treated with ketamine.

The expression of *Atp2b4* encoding PMCA4 isoform was reduced in GFP- and PMCA2b-expressing cells treated with ketamne (Figure 3F), and this effect was reproduced at the protein level (Figures 3G,H). It is reasoned that if ketamine-induced repression of PMCA4 was at least partially responsible for the prolongation of Ca^2+^ transients following KCl stimulation, then the heterologous expression of PMCA4 should facilitate Ca^2+^ recovery. To address this issue, PMCA4b-mCherry was transfected into H19-7 and effectively targeted the plasma membrane (Figure 3I). PMCA4b accelerated Ca^2+^ clearance in saline-treated GFP- (41 ± 5% vs. mock-transfected cells) and PMCA2b-expressing cells (30 ± 9% vs. mock-transfected cells), but in the latter case, it did not reach significance (Figure 3J, saline). Interestingly, co-expression of PMCA2b and PMCA4b, but not PMCA4b alone, was able to partially counterbalance ketamine-induced prolonged Ca^2+^ dynamics evoked by KCl and reduce t_1/2_ by 30 ± 5% vs. mCherry control (Figure 3J, ketamine).

### Association of PMCA2b-GFP to NMDAR1 Is Increased in the Presence of Ketamine

**Figure 4:**
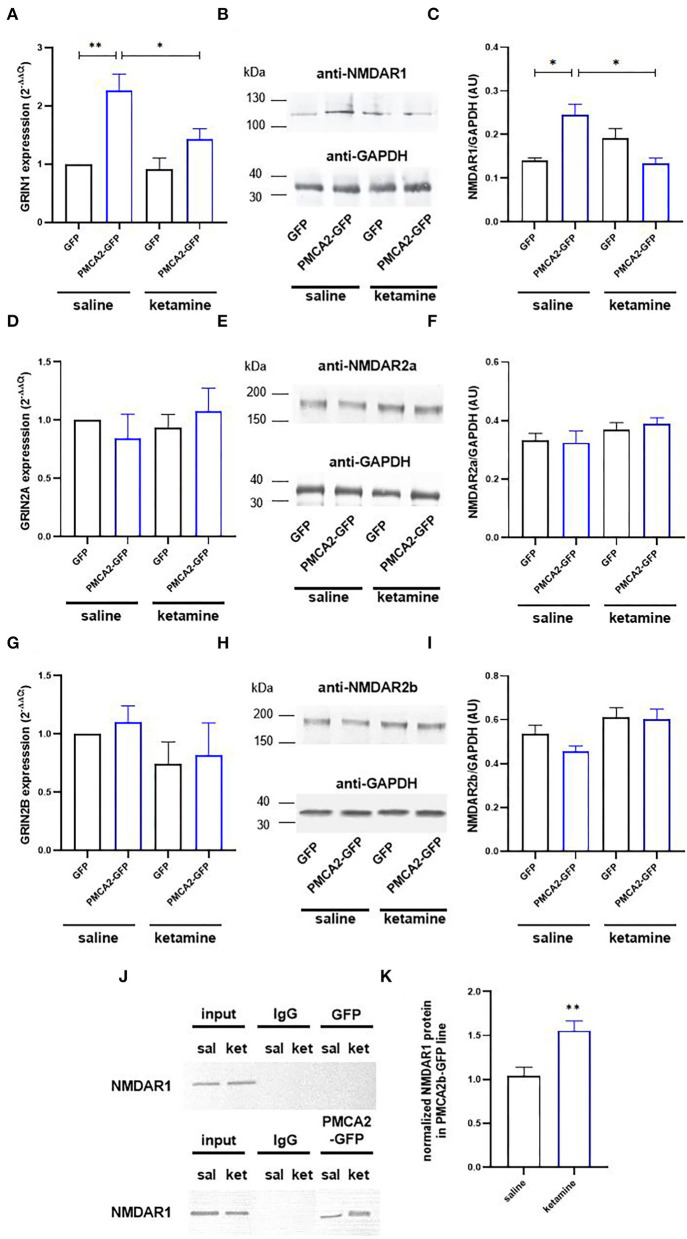
Ketamine enhances PMCA2b interaction with NMDAR1. **(A)** The expression of *Grin1* encoding NMDAR1 subunit evaluated with real-time PCR and calculated using the 2^−Δ*ΔCt*^ method. The expression level in GFP control was taken as 1, *n* = 4. **(B)** NMDAR1 protein level evaluated with the Western blot. GAPDH was taken as a loading control. The representative blots are presented. **(C)** Quantification of NMDAR1 protein level following normalization to endogenous Gapdh level, *n* = 3. AU, arbitrary units. **(D)** The mRNA and **(E)** protein levels of NMDAR2a. **(F)** Quantification of NMDAR2a protein level following normalization to endogenous Gapdh level, *n* = 3. **(G)** The mRNA and **(H)** protein levels of NMDAR2b. **(I)** Quantification of NMDAR2b protein level following normalization to endogenous Gapdh level, *n* = 3. **(J)** Co-immunoprecipitation of NMDAR1 and PMCA2b. Lysates from differentiating H19-7 cells were used to measure NMDAR1 protein level (input) in saline- and ketamine-treated conditions. Negative controls also included sepharose-linked secondary antibodies (IgG) and sepharose beads only. Representative blots are presented. **(K)** Quantitative densitometric analysis of band intensity in PMCA2b-expressing cells. The results are presented as arbitrary units defined as the optical density per mg protein, *n* = 3. *P < 0.05, **P < 0.01.

Because the potency of ketamine depends on the composition of NMDA receptor subunits (Dravid et al., 2007; Zorumski et al., 2016), we next checked the expression profiles of NMDAR1, NMDAR2a, and NMDAR2b. The expression of *Grin1* encoding the NR1 subunit was increased 2.2 ± 0.25 times in PMCA2b-GFP cells, but this effect was abolished by ketamine treatment (Figures 4A–C). No changes in mRNA and protein of *Grin2a* and *Grin2b* encoding subunits NR2a and NR2b, respectively, were seen in the presence or absence of ketamine (Figures 4D–I).

Co-immunoprecipitation results indicated that NMDAR1 was precipitated by anti-PMCA2 antibodies but not by the control IgG **(****Figure 4J**). Moreover, the NMDAR1/PMCA2 complex formation was more intense following ketamine treatment (Figure 4K).

### PMCA2b Modulates the Expression of Genes Involved in Glutamate and GABA Signaling

**Figure 5:**
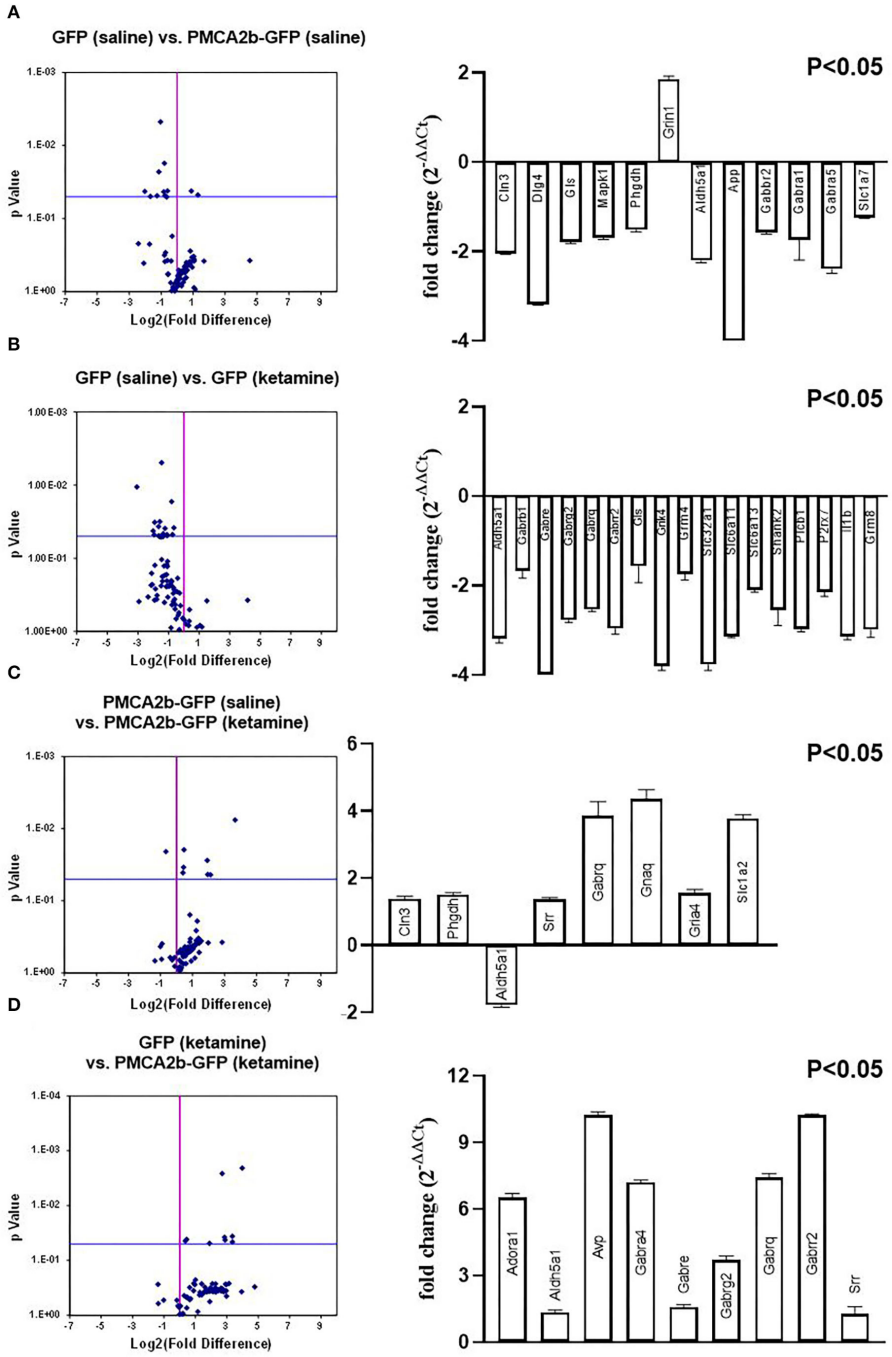
Microarray screening of fundamental genes involved in glutamate and GABA signaling. The SABiosciences rat microarray (PARN-152Z) was used to evaluate changes in the expression of genes involved in GABA and glutamate signaling. [**(A–D)**, left panel] Volcano plot analysis of expression changes calculated using company-provided software. [**(A–D)**, right panel] Genes whose expression was statistically significant (*P* < 0.05).

The NMDA receptors are well-known to be associated with both glutamatergic and GABAergic neurotransmission (Chen et al., 2000; Xue et al., 2011; Rajani et al., 2020). Moreover, our previous study demonstrated a link between PMCA2 expression and GABA metabolism (Kowalski et al., 2011). Therefore, in the next step, we performed a microarray screening of fundamental genes engaged in GABA and glutamate signaling to search for the changes at the level of transcription. The volcano plots (considering only those genes with *P* < 0.05) showed differences not only between GFP- and PMCA2b-GFP positive cells, but also differences between saline- and ketamine-treated cells (Figure 5). The transcripts of 11 genes were downregulated in response to PMCA2b expression in the absence of ketamine (Figure 5A), and 7 of these genes (*Dlg4, Mapk1, Slc1a2, App, Gabra1, Gabra5*, and *Grin1*) were specific to these conditions. In these conditions, the expression of Grin1 was increased by >2 times, which is consistent with real-time PCR results presented in Figure 4A. Treatment of GFP control with ketamine (Figure 5B) led to downregulation of 17 transcripts, and nine of these genes (*Gabrb1, Grik4, Slc32a1, Slc6a13, Shank2, Plcb1, P2rx7, Il1b*, and *Grm8*) were uniquely regulated. The opposite trend in the expression of analyzed genes was seen when PMCA2b-GFP data were compared before and after ketamine administration (Figure 5C) and between GFP- and PMCA2b-GFP positive cells both treated with ketamine (Figure 5D). When analyzing the effect of ketamine on PMCA2b-GFP expressing cells (Figure 5C), we identified higher expression of 2 unique genes (*Gria4* and *Gnaq*). Interestingly, the expression of PMCA2b-GFP in the ketamine model (Figure 5D) showed upregulation of 3 unique genes (*Adora1, Avp*, and *Gabra4*) and reversed the expression of *Aldh5a*. A detailed description of the analyzed genes is presented in Table 2.

**Table 2:** The list of genes with statistically significant (*P* < 0.05) expression change.

**Symbol**	**Description**	**RefSeq**	**Gene Name**
Adora1	Adenosine A1 receptor	NM_017155	–
Aldh5a1	Aldehyde dehydrogenase 5 family, member A1	NM_022851	Ssadh
App	Amyloid beta (A4) precursor protein	NM_019288	Abeta
Avp	Arginine vasopressin	NM_016992	DI, VP, Vas
Cln3	Ceroid-lipofuscinosis, neuronal 3	NM_0010069	–
Dlg4	Discs, large homolog 4 (Drosophila)	NM_019621	Dlgh4, PSD95, Sap90
Gabbr2	Gamma-aminobutyric acid (GABA) B receptor 2	NM_031802	Gpr51
Gabra1	Gamma-aminobutyric acid (GABA) A receptor, alpha 1	NM_183326	–
Gabra4	Gamma-aminobutyric acid (GABA) A receptor, alpha 4	NM_080587	–
Gabra5	Gamma-aminobutyric acid (GABA) A receptor, alpha 5	NM_017295	–
Gabrb1	Gamma-aminobutyric acid (GABA) A receptor, beta 1	NM_012956	GARB1
Gabre	Gamma-aminobutyric acid (GABA) A receptor, epsilon	NM_023091	–
Gabrg2	Gamma-aminobutyric acid (GABA) A receptor, gamma 2	NM_183327	–
Gabrq	Gamma-aminobutyric acid (GABA) receptor, theta	NM_031733	–
Gad1	Glutamate decarboxylase 1	NM_017007	Gad67
Gls	Glutaminase	NM_012569	Glut, RATGLUT
Gnaq	Guanine nucleotide binding protein (G protein), q polypeptide	NM_031036	Galphaq
Gria4	Glutamate receptor, ionotrophic, AMPA 4	NM_017263	GluA4, GluR-D, GluR4
Grin1	Glutamate receptor, ionotropic, N-methyl D-aspartate 1	NM_017010	GluN1, NMDAR1, NR1
Grik4	Glutamate receptor, ionotropic, kainate 4	NM_012572	GluK4, KA1
Grm4	Glutamate receptor, metabotropic 4	NM_022666	–
Grm8	Glutamate receptor, metabotropic 8	NM_022202	Glur8, Gprc1h, Mglur8, mGluR8b, mGlur
Il1b	Interleukin 1 beta	NM_031512	–
Mapk1	Mitogen activated protein kinase 1	NM_053842	Erk2
P2rx7	Purinergic receptor P2X, ligand-gated ion channel, 7	NM_019256	–
Phgdh	Phosphoglycerate dehydrogenase	NM_031620	–
Plcb1	Phospholipase C, beta 1 (phosphoinositide-specific)	NM_0010776	PLC'1, PLCbeta1, Phosphb
Shank2	SH3 and multiple ankyrin repeat domains 2	NM_133440	CortBP1, ProSAP1, Spank-3
Slc1a7	Solute carrier family 1 (glutamate transporter), member 7	NM_0011089	–
Slc32a1	Solute carrier family 32 (GABA vesicular transporter), member 1	NM_031782	Vgat, Viaat
Slc6a11	Solute carrier family 6 (neurotransmitter transporter, GABA), member 11	NM_024372	Gabt4, Gat3
Slc6a13	Solute carrier family 6 (neurotransmitter transporter, GABA), member 13	NM_133623	GAT-2
Srr	Serine racemase	NM_198757	–

### PMCA2b Expression Rescues Ketamine-Mediated Decrease in GABA Release *via* Inhibition of Its Metabolism by GABA-T

**Figure 6:**
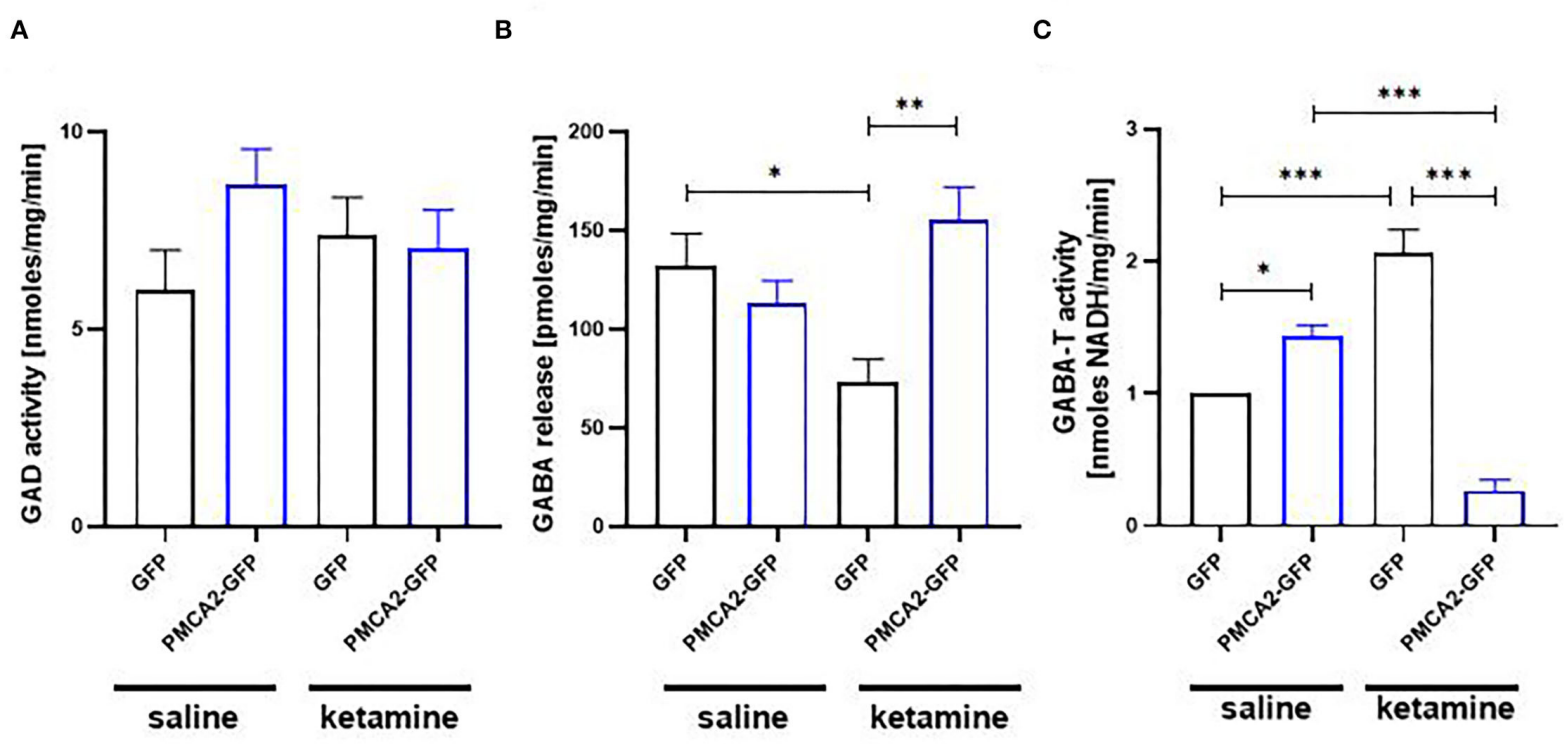
The effect of ketamine on GABA metabolism in the presence or absence of PMCA2b. **(A)** The activity of GAD assayed based on the fluorometric measurement of GABA and ninhydrin condensation product. *n* = 4. **(B)** Quantification of Ca^2+^-dependent GABA release over 5 min stimulation with 30 mM KCl done with GABA Elisa kit. *n* = 3. **(C)** The activity of GABA transaminase (GABA-T) expressed as NADH formation measured at 340 nm. *n* = 4. *P < 0.05, **P < 0.01, ***P < 0.001.

As glutamate and GABA metabolism converge at the level of glutamate decarboxylase (GAD), we next verified how PMCA2b-GFP expression in H19-7 cells affected GABA metabolism and secretion (Figure 6). No changes in the activity of GAD were detected between saline- and ketamine-treated cells (Figure 6A). To evaluate how PMCA2b and ketamine may affect GABA release, we mimicked the synaptic (Ca^2+^-dependent) release of this neurotransmitter (Figure 6B). Interestingly, ketamine decreased GABA release in GFP-expressing cells but not in PMCA2b-expressing cells. In line with that, we revealed increased activity of GABA transaminase (GABA-T) in GFP control treated with ketamine in relation to the saline-treated group (Figure 6C). By contrast, PMCA2b-GFP expressing cells treated with ketamine showed decreased activity of this enzyme.

## Discussion

Ketamine has been shown to affect the proliferation and differentiation of neural stem/progenitor cells (NSPCs). *In vitro* drug treatment at a concentration below the apoptotic threshold significantly inhibited the proliferation of rat fetal NSPCs and reduced the immunoreactivity of Ki67, a protein marker of cell proliferation (Dong et al., 2012; Wu et al., 2014). Similarly, perinatal exposure to ketamine *in vivo* inhibited the proliferation of neural stem cells in the ventricular and subventricular zones, two regions of intensive neurogenesis during embryonic development (Huang et al., 2015; Dong et al., 2016). The mechanistic explanation of this phenomenon is not entirely clear; however, several studies have suggested that it may involve ketamine's interference with neuronal Ca^2+^ homeostasis and toolkit. It is known that oscillatory Ca^2+^ signals establish a neuronal preference for undifferentiated neural crest cells (Carey and Matsumoto, 1999) and influence neurogenesis and proliferation in the embryonic rat ventricular zone (Owens and Kriegstein, 1998). Ketamine at a concentration of >300 μM has been shown to abolish Ca^2+^ oscillatory activity *in vitro* (Huang et al., 2013). Wu and colleagues (Wu et al., 2014) observed a dose-dependent decrease in a resting Ca^2+^ concentration as well as Ca^2+^-dependent inhibition of PKCα and ERK1/2 in neural stem cells isolated from the neonatal rat hippocampus. Bearing in mind the universality and fidelity of calcium signaling, it is not surprising that any alterations in subtle Ca^2+^ balance and Ca^2+^-dependent signaling may profoundly affect neurogenesis.

The significance of PMCA in the maintenance of neuronal Ca^2+^ homeostasis and the generation of discrete Ca^2+^ signals has been confirmed in various heterologous systems (Boczek et al., 2012; Pászty et al., 2015; Padányi et al., 2016; Calì et al., 2017). In line with others (Bhargava et al., 2002), we demonstrated the presence of PMCA1 and PMCA4 in differentiated H19-7 cells. The expression of PMCA2b-GFP in these cells did not affect resting [Ca^2+^]_c_ but reduced both the amplitude of KCl-evoked Ca^2+^ influx and the halftime of Ca^2+^ signal decay, but only in the absence of ketamine. The drug significantly reduced Ca^2+^ influx during KCl-induced depolarization but markedly delayed Ca^2+^ clearance irrespective of the presence of PMCA2b. PMCA2b exhibits very high basal activity, hence it can quickly respond to even subtle changes in [Ca^2+^]_c_. Overexpression of this isoform has been demonstrated to rapidly clear SOCE-mediated Ca^2+^ transients (Pászty et al., 2015) and reduce KCl-induced increases in [Ca^2+^]_c_ (Jiang et al., 2010). Our previous study showed that PMCA isoforms can be equally inhibited by ketamine when interacting with the enzyme at two potential sites: the catalytic loop and the C-terminal domain (Boczek et al., 2015b). A direct consequence of such inhibition was a dramatic reduction in Ca^2+^ pumping activity, which may at least in part explain the lack of PMCA2b effect on the rate of Ca^2+^ clearance in H19-7 cells treated with ketamine.

It is striking that PMCA repression would be sufficient to generate prolonged Ca^2+^ transients in differentiated H19-7 cells. This is clearly visible for PMCA4, which was repressed by ketamine, but its overexpression enhanced the rate of Ca^2+^ clearance following KCl depolarization. Similarly, we observed an increase in the halftime of decay in PMCA1-depleted GFP control cells, suggesting an important contribution of this isoform to Ca^2+^ clearance following KCl stimulation. In contrast, PMCA1 depletion by siRNA did not affect t_1/2_ in ketamine-treated cells. In contrast to resting conditions, when PMCA levels are in excess toward the substrate, during evoked transients one may expect a limitation of Ca^2+^ extrusion when PMCA1 is depleted. Our results indicate that the ketamine concentration used here was sufficient to effectively inhibit PMCA activity. In such circumstances, Ca^2+^ clearance may be conferred by other systems such as the Na^+^/Ca^2+^ exchanger or SERCA pump being slower than PMCA. Co-transfection of PMCA2b and PMCA4b was able to partially rescue reduced Ca^2+^ clearing potency putatively because the inhibitory effect of the drug was overcome. This could also suggest that the PMCA2b/PMCA4b ratio may control ketamine's effect on calcium extrusion in our experimental model.

Yet, there is also another interesting observation regarding the heterologous expression of PMCA2b in H19-7. We demonstrated concomitant upregulation of PMCA1 that persisted after ketamine treatment and significant drug-dependent downregulation of PMCA4 regardless of the presence of PMCA2b. Changes in PMCA isoform expression (PMCA1, PMCA3, and PMCA2 in particular) during neuronal maturation have been demonstrated to be Ca^2+^-dependent (Kip et al., 2006; Sepúlveda et al., 2007; Mata and Sepulveda, 2010; Krebs, 2015). In contrast, during the maturation of hippocampal neurons *in vitro*, the expression of PMCA4 was downregulated in response to prolonged KCl stimulation (Kip et al., 2006). These changes, coinciding with a shift of splice variants from b-type to a-type, are believed to be required for the functional specialization of Ca^2+^ signaling in developing neurons, which becomes important for the induction of early neural genes and later axon pathfinding and synapse formation (Zheng, 2000; Michaelsen and Lohmann, 2010; Toth et al., 2016).

As PMCA isoforms appear in a defined time frame during neuronal development, expression of PMCA2 before day E18 may produce “more mature” neurons. The morphological features that can be ascribed to early neuritogenesis are already seen in PMCA2b-expressing non-differentiated cells and further during differentiation. This may induce earlier upregulation of PMCA1 by a mechanism controlling its gene transcription, for instance, through c-myc-dependent regulation. It has been demonstrated, albeit, in non-neuronal cells, that ketamine decreased the expression of c-myc (Hu et al., 2020), which is known to repress PMCA1 (Afroze and Husain, 2001). However, other post-transcriptional or post-translational events such as mRNA stability and/or translation efficiency may also play a role. The downregulation of PMCA4 by ketamine was seen in both the GFP and PMCA2b-GFP groups, suggesting a mechanism independent of the presence of PMCA2b. Possible explanations may be the ketamine-mediated generation of reactive oxygen species (Behrens et al., 2007) targeting PMCA4-rich lipid rafts, attenuation of c-Fos and c-Jun expression, and subsequent inhibition of AP-1 transcription factor (Lerea et al., 1992; Lisek et al., 2020), or the involvement of calcineurin, which was previously demonstrated during maturation of cerebellar granule cells (Guerini et al., 2000). Interestingly, our data did not show neuronal retraction or changes in the level of differentiation markers in the presence of ketamine. Considering inhibition of PMCA by ketamine (Boczek et al., 2015b), more intense differentiation of PMCA2b-expressing cells may not be directly related to PMCA pumping activity and its role in the restoration of basal [Ca^2+^]_c_. Instead, the expression of this isoform can create a scaffold for the interaction of several PMCA2-associated signaling molecules. A Ser/Thr protein phosphatase calcineurin has been demonstrated to strongly interact with PMCA2 (Holton et al., 2007) and be directly involved in the outgrowth of embryonic axons (Graef et al., 2003).

It has been well-established that the population of GABAergic neurons is formed before glutamatergic neurons during hippocampal development (Soriano et al., 1986). In the immature brain, GABA is the main excitatory neurotransmitter and has trophic effects on neuronal development (Luján et al., 2005; Cheung and Yew, 2019). Given this importance, any alterations in GABAergic transmission during early development may have profound consequences for adult brain function. Several reports have demonstrated the developmental consequences of NMDA receptor antagonism on GABAergic neurons, including reduced density (Abekawa et al., 2007), alterations in dendritic growth and dendritic arbor architecture (Vutskits et al., 2006), long-term defects in dendrite number and spine morphology (Aligny et al., 2014), and enhanced apoptotic death of GABAergic precursors (Aligny et al., 2014). The mechanism by which ketamine affects the GABAergic cell population is far from being elucidated. Our study revealed that this effect involves significant ketamine-mediated downregulation of GABA(A) receptor subunits as well as GABA transporters. GABA(A) receptors fulfill a critical role in functional and structural GABAergic synapse development (Fuchs et al., 2013; Oh et al., 2016). However, the proper maturation of GABAergic interneurons requires the activity of NMDA receptors (Gu et al., 2016; Hanson et al., 2019). It has been demonstrated that both proteins co-localize in early development (Cserép et al., 2012), where GABA(A) receptor activation can remove Mg^2+^ blockage of the NMDA receptor, activating it (Wang and Kriegstein, 2008). Hence, not only GABA(A) but also functional NMDA receptors are expected to correctly balance excitation and inhibition in differentiating neurons. In the early stages of development, proper GABA release and its signaling through the GABA receptor have been demonstrated to control DNA synthesis in progenitor cells and thus reduce the total number of these cells (Loturco et al., 1995). In view of that, ketamine inhibiting the NMDA receptor and influencing the transcriptional control of GABA(A) receptor in progenitor cells may interfere with developmental cell death and result in a higher number of newly generated neurons in the rat hippocampus as shown previously (Keilhoff et al., 2004). In contrast, ketamine has been demonstrated to destabilize the growth of dendritic spines in developing hippocampal neurons (Jiang et al., 2018) and the elimination of the early excitatory GABA(A)-dependent signal was detrimental to neuronal morphology (Cancedda et al., 2007).

The exact mechanism by which PMCA2b restricts ketamine-induced apoptosis of hippocampal progenitor cells remains elusive, but our data indicate it may involve a Bad-dependent block of apoptosis initiation. Bad is known to be dephosphorylated by calcineurin, allowing it to bind and inhibit the anti-apoptotic Bcl-2 and Bcl-xL (Wang et al., 1999). PMCA2 binds calcineurin and recruits it to low Ca^2+^ plasma membrane microdomains, thereby reducing its phosphatase activity (Holton et al., 2010). The formation of the PMCA2b/calcineurin inhibitory complex may be a critical step in determining apoptosis in the presence of ketamine. Based on our microarray data, it is also tempting to speculate about the homeostatic compensation of GABA(A) receptor subunit expression to ketamine. However, the differences in the microarray profile suggest different responses of progenitor cells to ketamine in the presence of PMCA2b. These compensatory changes may be a part of a more complex adaptive mechanism aimed in response to a ketamine-mediated decrease in GABA release, which is known to act in a paracrine fashion in early neuronal development (Manent and Represa, 2007). So far, up to 19 different GABA(A) receptor subunits have been identified, but the physiological role of many of them has not been studied (Sieghart and Sperk, 2002). The increased expression of particular subunits, as shown in our microarray screening, may affect GABA(A) receptor assembly and lead to the formation of physiologically and/or pharmacologically distinct receptor subtypes. Although it is difficult to speculate about the functional consequences of these changes, it is apparent from our study that early PMCA2b expression changes the profile of GABA(A) receptor subunits, which may affect apoptosis during differentiation of progenitor cells in the presence of ketamine. However, the influence on the expression of other genes involved in GABAergic signaling cannot be ruled out. For instance, strong upregulation of Adora1 encoding adenosine A1 receptor has been revealed in response to PMCA2b expression in the presence of ketamine. Several recent reports have demonstrated this receptor to be neuroprotective (Gholinejad et al., 2018; Xiao et al., 2019; Zhang et al., 2020). In adult brain, treatment with ketamine was associated with intensified formation of the PMCA/PSD-95/NMDAR1 complex (Lisek et al., 2017), which is thought to tether PMCA to the site of massive Ca^2+^ influx. In differentiating progenitor cells, we observed ketamine-evoked increased co-immunoprecipitation of PMCA2b with NMDAR1. This may constitute an adaptive mechanism aimed to counterbalance abnormal Ca^2+^ elevations, thus preventing from Ca^2+^-induced neuronal apoptosis.

Here, we showed that H19-7 progenitor cells possess the cellular machinery that is necessary for GABA synthesis and its Ca^2+^-dependent secretion. Despite unaffected GAD activity, Ca^2+^-dependent GABA release was diminished by ketamine, but this effect was reversed by PMCA2b expression. One possible explanation is the decreased activity of GABA-T in ketamine-treated PMCA2b-expressing cells presented here, which is expected to reduce the rate of GABA degradation, thus making it available for neurosecretion. Interestingly, the activity of GABA-T seems to depend on the activity of PMCA, as the inhibition of PMCA by ketamine also diminished the activity of GABA-T. As mitochondrial GABA-T is sensitive to pH in the matrix (Buu and Van Gelder, 1974), one of the possible mechanisms may involve Ca^2+^- and PMCA-dependent mitochondrial acidification or changes in mitochondrial membrane potential (Poburko et al., 2011; Boczek et al., 2014), an effect that is dissipated when PMCA pumping activity is blocked by ketamine. However, other mechanisms involving the interference of ketamine with PMCA binding partners and/or the effect of PMCA inhibition on mitochondrial Ca^2+^ accumulation cannot be ruled out.

In mature neurons, PMCA2b localizes to synaptic spines where it colocalizes with a variety of synapse-associated proteins, including SAP90/PSD95, SAP97, and chapsyn110/PSD93 and SAP102, many of them forming scaffolds for signaling molecules (Kim et al., 1998; DeMarco and Strehler, 2001). At synapses, PMCA2 also co-exists with syntaxin (Garside et al., 2009), which associates with SNAP-25 and synaptobrevin to form a part of the SNARE complex responsible for neurotransmitter exocytosis (Sørensen, 2005). In neurosecretory PC12 cells, PMCA2 has been shown to affect synaptobrevin expression through calcineurin/NFAT signaling and interfere with the assembly of the SNARE complex during dopamine release (Kosiorek et al., 2014). Therefore, the effect of PMCA2b expression on GABA release machinery cannot be ruled out as well.

## Conclusion

Using hippocampal progenitor cells, we showed that expression of PMCA2b early in development is sufficient to induce a neuritogenesis-like phenotype in the absence of any external stimuli and partially protect differentiating cells from ketamine-induced neurotoxicity. This protection seems to be conferred by PMCA2b-dependent control of genes involved in GABAergic and glutamatergic signaling, as well as intensified PMCA2b/NMDAR1 clustering putatively to prevent abnormal Ca^2+^ rises and Ca^2+^-dependent apoptosis. We also showed that PMCA2b expression prevented ketamine-induced GABA impairments *via* a mechanism of GABA-T inhibition. Our results also demonstrated that manipulations in the Ca^2+^ signaling toolkit may have profound influences on the course of neuronal differentiation. The time frame of embryonic exposure to such manipulations may be one of the most important determinants of molecular defects later in development.

## Data Availability Statement

The original contributions presented in the study are included in the article/supplementary materials, further inquiries can be directed to the corresponding author/s.

## Author Contributions

ML, JM, MS, BF, and FG performed experiments and analyzed the data. TB and LZ wrote and edited the manuscript. All authors contributed to the article and approved the submitted version.

## Funding

This study was supported by the National Science Centre (Narodowe Centrum Nauki) grant no. 2020/39/D/NZ4/01250 and by the Medical University of Lodz grant no. 503/6-086/-2/503-61-001.

## Conflict of Interest

The authors declare that the research was conducted in the absence of any commercial or financial relationships that could be construed as a potential conflict of interest.

## Publisher's Note

All claims expressed in this article are solely those of the authors and do not necessarily represent those of their affiliated organizations, or those of the publisher, the editors and the reviewers. Any product that may be evaluated in this article, or claim that may be made by its manufacturer, is not guaranteed or endorsed by the publisher.
